# Deciphering non-canonical ubiquitin signaling: biology and methodology

**DOI:** 10.3389/fmolb.2023.1332872

**Published:** 2024-02-13

**Authors:** Nila K. van Overbeek, Tim Aguirre, Gerbrand J. van der Heden van Noort, Blagoy Blagoev, Alfred C. O. Vertegaal

**Affiliations:** ^1^ Department of Cell and Chemical Biology, Leiden University Medical Center, Leiden, Netherlands; ^2^ Department of Biochemistry and Molecular Biology, University of Southern Denmark, Odense, Denmark

**Keywords:** ubiquitin, non-canonical ubiquitination, oxyester, thioester, proteomics, affinity purification, mass spectrometry, E3 ligase

## Abstract

Ubiquitination is a dynamic post-translational modification that regulates virtually all cellular processes by modulating function, localization, interactions and turnover of thousands of substrates. Canonical ubiquitination involves the enzymatic cascade of E1, E2 and E3 enzymes that conjugate ubiquitin to lysine residues giving rise to monomeric ubiquitination and polymeric ubiquitination. Emerging research has established expansion of the ubiquitin code by non-canonical ubiquitination of N-termini and cysteine, serine and threonine residues. Generic methods for identifying ubiquitin substrates using mass spectrometry based proteomics often overlook non-canonical ubiquitinated substrates, suggesting that numerous undiscovered substrates of this modification exist. Moreover, there is a knowledge gap between *in vitro* studies and comprehensive understanding of the functional consequence of non-canonical ubiquitination *in vivo*. Here, we discuss the current knowledge about non-lysine ubiquitination, strategies to map the ubiquitinome and their applicability for studying non-canonical ubiquitination substrates and sites. Furthermore, we elucidate the available chemical biology toolbox and elaborate on missing links required to further unravel this less explored subsection of the ubiquitin system.

## 1 Introduction to the ubiquitin system

To adapt to environmental changes and regulate proteostasis, cells remodel their proteome via post-translational modifications (PTMs). PTMs fine-tune protein function, localization, stability and protein-protein interactions, enabling cellular responses. Ubiquitination is a PTM initially recognized for its role in proteasome-mediated degradation, which was acknowledged with the Nobel prize in Chemistry granted to Aaron Ciechanover, Avram Hershko and Irwin Rose in 2004 (Hershko et al., 1980; Hershko et al., 2000). It is now clear that this abundant and highly versatile 76-amino acid protein is tightly regulating numerous cellular processes, also beyond protein degradation.

**FIGURE 1:**
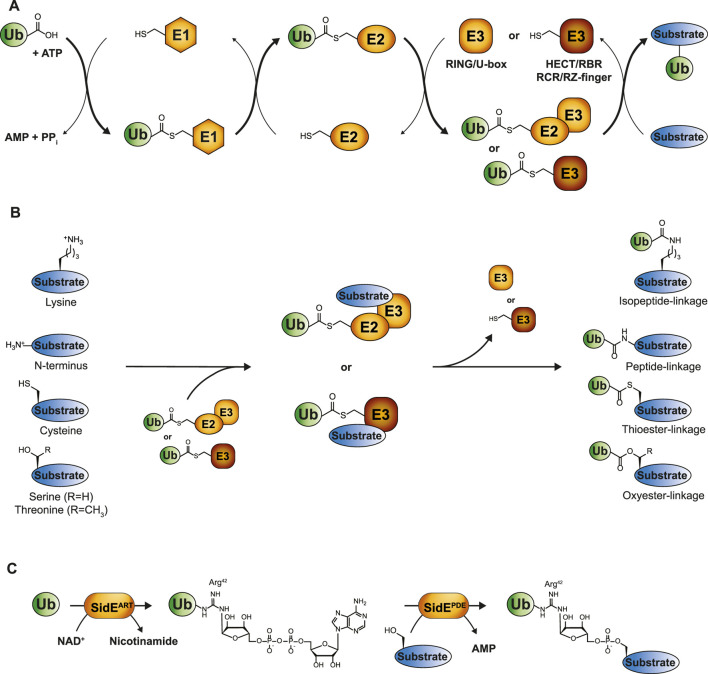
The ubiquitin cascade. **(A)** Ubiquitin is transferred to its substrates by a three-step enzymatic cascade involving E1 activating enzymes, E2 conjugating enzymes and E3 ligases. RING and U-box E3s coordinate the direct transfer of ubiquitin from E2 to the designated substrate, while transthiolating E3s covalently bind ubiquitin via their own catalytic cysteine. The final ubiquitination step occurs through a nucleophilic attack of the substrate on the electrophilic thioester-carbonyl of the E2-or E3-ubiquitin conjugate. **(B)** Depending on the substrate, these nucleophiles comprise lysine side chains, protein N-termini or side chains from unconventional amino acids such as cysteine, threonine or serine, thereby giving rise to isopeptide-, peptide-, thioester, or oxyester-linkages, respectively. **(C)** Phosphoribosyl-linked serine ubiquitination by *Legionella pneumophila* is mediated by the ADP ribosyl transferase (ART) and phosphodiesterase (PDE) domains of a single SidE family member. SidE^ART^ first ADP-ribosylates ubiquitin’s Arg42 through the transfer of ADPR from NAD^+^ to ubiquitin. Subsequently, the SidE PDE domains recognize this intermediate and catalyze the formation of a phosphodiester bond between ADP-ribosylated ubiquitin and a serine residue in the substrate protein.

The process of ubiquitin conjugation entails an enzymatic cascade consisting of activating E1-, conjugating E2-and ligating E3-enzymes. First, the C-terminus of ubiquitin is attached to the catalytic cysteine of the E1 enzyme via a thioester bond at the expense of ATP, after which the activated ubiquitin is passed to the catalytic cysteine residue of the E2 in the form of an E2-ubiquitin thioester intermediate. The E3 enzyme typically conjugates ubiquitin’s C-terminal glycine to the ε-amino group of lysine residues in a substrate by the formation of an isopeptide bond (Figures 1A, B) (Hershko and Ciechanover, 1992; Hershko et al., 1983). Strict control of ubiquitination is achieved by the coordinated action of two E1 enzymes, approximately 40 E2 enzymes and more than 600 E3 enzymes, ensuring spatiotemporal specificity (Pickart, 2001; Clague et al., 2015).

Canonical ubiquitin modification occurs on lysine residues of substrates, giving rise to mono- or multi-ubiquitination. Ubiquitin’s own N-terminus or one of its seven lysine residues can also undergo ubiquitination, forming polymeric ubiquitin chains that exhibit various conformations such as homotypic, mixed or branched [reviewed in (Komander and Rape, 2012; Perez Berrocal et al., 2019; Swatek and Komander, 2016; Yau and Rape, 2016)]. This greatly expands the complexity of ubiquitin signaling. Ubiquitination is a reversible and dynamic modification and around 100 deubiquitinating enzymes (DUBs) can cleave off individual ubiquitin molecules from substrates or break down ubiquitin chains, enabling ubiquitin recycling (Komander et al., 2009).

There is a continuous discovery of additional layers of versatility and complexity of the ubiquitin system that broaden the scope of ubiquitin signaling beyond the established pathways. Here, we will focus on one such phenomenon: non-canonical ubiquitination. We will discuss the current knowledge of non-canonical ubiquitination, review methods to identify ubiquitin substrates and how these can be employed to expand our knowledge on non-canonical ubiquitination substrates and sites. Lastly, we will share future considerations of non-canonical ubiquitination and discuss (bio)chemical-tools that can aid to decipher how non-canonical ubiquitination distinctly regulates the proteome.

## 2 The rise of non-canonical ubiquitination—expanding the ubiquitin code

The first observation of lysine-independent ubiquitination dates back to 2005 (Cadwell and Coscoy, 2005; 2008; Wang et al., 2007; Herr et al., 2009). Emerging evidence has since indicated the presence of ubiquitination events that extend beyond lysine residues, which are commonly referred to as “non-canonical ubiquitination.” However, they remain scarcely described for substrates in comparison to the multitude of lysine ubiquitination examples. Non-canonical ubiquitination comprises the formation of a chemical bond distinct from the isopeptide bond that typically links ubiquitin to a lysine residue of the substrate. These encompass peptide bonds between ubiquitin and the α-amino group of the N-terminus of substrates, thioester-based linkages between ubiquitin and cysteine residues, and oxyester bonds, where ubiquitin is conjugated to serine or threonine residues (Figure 1B) (McDowell and Philpott, 2016). Tyrosine could, in theory, also establish an oxyester bond with ubiquitin, although no *in vivo* examples of tyrosine ubiquitination in eukaryotes have been reported to date. Pathogens have developed unique forms of ubiquitination, even expanding the non-canonical ubiquitination observed in eukaryotes. The prime example is *Legionella pneumophila*, linking substrate protein and ubiquitin via an unusual phosphoribosyl-linkage (Box 1), which has recently been reviewed (Dikic and Schulman, 2023).

Most of the documented cases of non-canonical ubiquitination were stumbled upon during research on specific proteins, often involving recombinant enzymes or artificial substrates, rather than identified during systematic investigation of non-canonical ubiquitination itself. Non-canonical ubiquitination is still often overlooked within the ubiquitin research field, but should be carefully considered based on the biological significance. Many substrates presumably remain undiscovered, emphasizing the need for unbiased high-throughput studies to capture the broad range of non-canonical ubiquitination events and for the development of an appropriate toolbox to study their dynamics.

### 2.1 N-terminal ubiquitination

In addition to modifying ε-amino groups of lysine residues, ubiquitin can also be conjugated to the N-terminal amino group of target proteins. The observation that ubiquitin-mediated-proteasome-dependent degradation of a lysine-deficient MyoD was unaffected, but chemical modification of the first α-amino group did abolish ubiquitination, brought forth this concept of N-terminal ubiquitination (Breitschopf et al., 1998). Functional relevance of this modification has since been demonstrated for numerous substrates [summarized in (Ciechanover and Ben-Saadon, 2004; McDowell and Philpott, 2016)]. For instance, N-terminal ubiquitination was shown to target proteins such as Ngn2 (McDowell et al., 2010), p14ARF (Liu et al., 2022) and p21 (Bloom et al., 2003; Coulombe et al., 2004) for degradation, and to distinctly alter catalytic activity of the DUBs UCHL1 and UCHL5 (Davies et al., 2021). Moreover, N-terminal ubiquitination delayed aggregation of amyloid proteins associated with neurodegenerative disorders (Ye et al., 2020).

For now, only few studies have reported single E2 and E3 enzymes capable of facilitating N-terminal ubiquitination. Its flexible C-terminus enables UBE2W to selectively ubiquitinate α-amino groups of N-termini (Scaglione et al., 2013; Tatham et al., 2013; Vittal et al., 2015). Furthermore, one study suggests that the E3 ligase HUWE1 can catalyze N-terminal ubiquitination of the aforementioned MyoD. Although it does so only for lysine-less MyoD and not for wild type MyoD (Noy et al., 2012), questioning the *bona fide* esterification activity of HUWE1 *in vivo*.

However, current knowledge of the broad range of substrates prone to N-terminal ubiquitination suggests the presence of a solid ubiquitin conjugation machinery capable of forming these peptide bonds (Akimov et al., 2018a; Akimov et al., 2018b).

### 2.2 Non-lysine ubiquitination

Since the initial discovery of non-lysine ubiquitination by viral E3 ligases, research has now revealed numerous enzymes and substrates involved in this modification (Table 1). The viral E3 ligases MIR1 and MIR2 were the first to be identified as modifiers of cysteine residues in the cytosolic tail of MHC I (Cadwell and Coscoy, 2005; Cadwell and Coscoy, 2008), whereas mK3 was shown to ubiquitinate serine or threonine residues within this MHC I tail (Wang et al., 2007; Herr et al., 2009). Complementary, the authors identified an E2 enzyme, UBEJ2J, that could cooperate with mK3 to ubiquitinate serine or threonine residues of MHC I (Wang et al., 2009). This ester bond forging activity of UBE2J2 has also been observed for its yeast homolog Ubc6. Notably, the identification of serine auto-ubiquitination of Ubc6 provided the first mass spectrometric validation of non-lysine ubiquitination (Weber et al., 2016).

**TABLE 1:** Overview of documented ubiquitination of cysteine (C), serine (S) and threonine (T) residues. Indicated, when known, are the “writers” (E2 and E3 enzymes catalyzing the ubiquitination), the substrate and the functional consequence of the ubiquitination. Non-canonical ubiquitination of MARCH-1 was detected using a lysine-less protein, the term “non-lysine” indicates that the residue(s) undergoing ubiquitination remains unspecified.

Type	Writers (E2/E3)	Substrate	Outcome	References
S, T	Ubc6 (yeast E2, homolog of UBE2J2)/Doa10 (yeast E3, homolog of MARCH6)	Asi2	Degradation	Boban et al. (2015), Weber et al. (2016)
C, S, T		BID	Degradation	Tait et al. (2007)
S, T	VPU-mediated (viral protein); SCF^β−TrCP^ (E3)	CD4 and BST-2	Degradation	Magadán et al. (2010), Tokarev et al. (2011)
S	ARIH1 (E3)	CCNE		Purser et al. (2023)
S, T		hD4R		Skieterska et al. (2015), Peeler et al. (2017)
S, T	HOIL-1 (E3 ligase)	IRAK4, IRAK2, MyD88, ubiquitin	Regulation of cytokine signaling	Kelsall et al. (2019), Petrova et al. (2021), Rodriguez Carvajal et al. (2021), McCrory et al. (2022)
Non-lysine	UBE2D1 (E2)	MARCH-1		Lei et al. (2018)
S, T	UBE2J2 (E2)/mK3 (viral E3)	MHC-1	ER-associated degradation	Wang et al. (2007), Herr et al. (2009), Wang et al. (2009)
C	MIR1, MIR2 (viral E3s)	MHC-1	Lysosomal degradation	Cadwell and Coscoy, 2005 (2008)
C, S, T		Ngn2	degradation	Vosper et al. (2009), McDowell et al. (2010)
S, T	MYCBP2 (E3)	NMNAT2		Pao et al. (2018)
S, T	HRD1 (E3)	NS-1	ER-associated degradation	Shimizu et al. (2010)
S, T		NY-ESO-1 (antigen)		Golnik et al. (2016)
C	Pex4p (yeast E2)	Pex18p (yeast)	Recycling	Hensel et al. (2011), El Magraoui et al. (2013)
C	Pex4p (yeast E2), UBE2D1/2/3 (mammalian E2)	Pex5p (yeast and mammalian)	Recycling	Carvalho et al. (2007), Williams et al. (2007), Grou et al. (2008), Okumoto et al. (2011), Schwartzkopff et al. (2015)
C	Pex4p (yeast E2), Pex2p/Pex10p/Pex12p (yeast E3s)	Pex20p	Recycling	Léon and Subramani (2007), Liu and Subramani (2013)
C	March6 (E3)	SQLE	Degradation	Chua et al. (2019)
S	HRD1 (E3)	TCRα	ER-associated degradation	Ishikura et al. (2010)

Beyond viral E3 ligases, different types of assays have since enabled the identification of cellular E3 ligases engaging in non-lysine ubiquitination. By utilizing activity-based protein profiling and biochemical assays, Pao and others showed esterification activity for the E3 ligase MYCBP2 (Pao et al., 2018). HRD1 was found to ubiquitinate NS-1, an ER-associated degradation (ERAD) substrate, predominantly on serine or threonine residues and only mutation of lysine, serine and threonine could stabilize NS-1 (Shimizu et al., 2010). Consistently, ubiquitination was abolished upon NaOH treatment, which hydrolyzes ester-linked ubiquitin and leaves isopeptide bonds intact. Interestingly, other ubiquitinated ERAD substrates were also sensitive to elevated pH values, indicating a broader prevalence of oxyester linkages (Shimizu et al., 2010).

Among the cellular E3-ligases, the esterification activity of HOIL-1, a regulator of inflammation, is more broadly studied. Both *in vitro* and *in vivo* data showed oxyester-linked ubiquitination activity of HOIL-1 for three substrates involved in cytokine production: IRAK2, IRAK4 and MyD88. Interestingly, all three substrates exhibited lysine ubiquitination as well (Kelsall et al., 2019; Rodriguez Carvajal et al., 2021; McCrory et al., 2022), suggesting potential interplay between lysine and non-canonical ubiquitination in regulating intensity and duration of cytokine signaling (Petrova et al., 2021). HOIL-1 belongs to the class of RING-in-between-RING (RBR) ligases and very recent findings showed serine ubiquitination of a CCNE peptide by ARIH1—another RBR E3 ligase. ARIH1 also demonstrated ubiquitination of both serine and lysine residues (Purser et al., 2023). Furthermore, functional interplay between lysine- and cysteine ubiquitination plays a well-established role in regulating peroxisomal import proteins. They are responsible for the translocation of other proteins to the peroxisomal matrix by cycling between the peroxisome and the cytoplasm. Cycling of transporter proteins Pex5p, Pex18p and Pex20p is tightly regulated by ubiquitination of a conserved cysteine residue, whereas lysine poly-ubiquitination of these proteins targets them for proteasomal degradation (Léon et al., 2006; Léon and Subramani, 2007; Platta et al., 2007; Williams et al., 2007; Hensel et al., 2011; Okumoto et al., 2011; Liu and Subramani, 2013; Schwartzkopff et al., 2015; Pedrosa et al., 2023).

To dynamically regulate this non-canonical ubiquitin signaling, “erasers” of ubiquitin are required to specifically cleave non-isopeptide bonds. By subjecting six DUBs (USP2, USP7, USP15, OTUB1, OTUB2 and UCHL3) to a cleavage assay utilizing ester- and isopeptide-linked conjugates, Sun and others were able to show esterase activity of USP2, USP7 and USP15 (Sun et al., 2018). The panel of DUBs capable of cleaving ester linkages was further expanded by a DUB activity assay screening 53 recombinant DUBs. The high-throughput assay, using ester linkages formed by MYCBP2, revealed several classes of DUBs proficient in cleaving the ester linkages. Apart from two exceptions (TRABID and the viral vOTU), the OTU class lacked esterase activity, whereas the USP and UCH families of DUBs cleaved both isopeptide- and oxyester linkages with similar kinetics. Interestingly, the MJD family of DUBs exhibited high selectivity towards serine and threonine linkages with comparable catalytic efficiency for threonine- and isopeptide linkages by the MJD member JOSD1 (De Cesare et al., 2021). Moreover, esterase activity of the USP family member USP9X has been observed in a cellular context whereby hydrolyzing the ubiquitin thioester bonds in the aforementioned Pex5p initiated new translocation cycles of peroxisomal matrix proteins (Grou et al., 2012). Thus, non-canonical ubiquitination leverages ubiquitin machinery components with esterification and esterase activity to form and cleave specific bonds linking ubiquitin to cysteine, serine or threonine residues.

Lastly, the ability of ubiquitin to form lysine-linked polyubiquitin chains is one the most important characteristics of this PTM, giving rise to the grand diversity of ubiquitin signals. These ubiquitin polymers are probably more complex than previously thought, since recent studies have reported internal ubiquitination of serine and threonine residues, indicating the existence of ester-linked ubiquitin chains (Kelsall et al., 2019; Rodriguez Carvajal et al., 2021; McCrory et al., 2022). More specifically, *in vitro* ubiquitination assays showed Thr12, Ser20, Thr22 and Thr55 linked ubiquitin polymers (Kelsall et al., 2019; Rodriguez Carvajal et al., 2021), while Thr12, Thr14, Ser20 and Thr22 linkages have also been found in cell lysates (McCrory et al., 2022). This again illustrates there is still more to discover and integrate into our current overview of ubiquitin signaling mechanisms.

Research on non-canonical ubiquitination [reviewed in (McDowell and Philpott, 2016; McClellan et al., 2019; Kelsall, 2022; Squair and Virdee, 2022; Dikic and Schulman, 2023)], has mostly been based on *in vitro* studies using recombinant proteins. These studies exploit sensitivity of ester-bonds to alkaline conditions or use mutated proteins devoid of their lysine residues. They are thus limited in revealing the full spectrum of non-canonical ubiquitin modifications and their specific sites. Moreover, non-canonical ubiquitination of lysine-less proteins does not disclose whether the serine, cysteine or threonine residues are preferentially ubiquitinated over lysine residues, and therefore possibly represent *in vitro* artefacts.

## 3 Mapping the ubiquitin landscape

**FIGURE 2:**
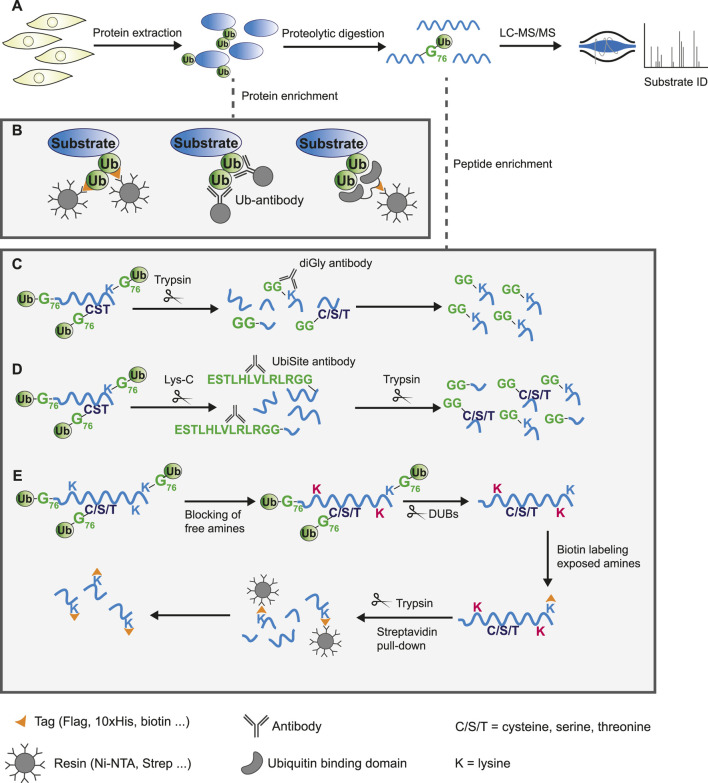
Protein or peptide based enrichment strategies to identify the ubiquitinome by mass spectrometry analysis. **(A)** General workflow of mass spectrometry based proteomics experiment. Proteins are extracted from cells or tissue and digested into peptides using proteases. These peptides are separated by liquid-chromatography (LC) and analyzed by tandem mass spectrometry (MS/MS) to allow for protein identification based on fragmented ion spectra. Due to low abundance of ubiquitinated substrates in the total proteome, modified proteins are commonly enriched either on a protein- or peptide-based level. **(B)** Schematic overview of ubiquitin substrate enrichment strategies at a protein level. Exogenous expression or endogenous tagging of ubiquitin followed by purification of modified substrates from cell lysates using anti-epitope antibody conjugated beads (left). Pull-down of ubiquitinated proteins by immunoprecipitation using specific ubiquitin antibodies (middle). Epitope tagged ubiquitin-binding domains bind ubiquitin with high affinity and enrich substrates through pull-down with anti-epitope antibody conjugated beads (right). **(C–E)** Schematic overview of approaches to enrich ubiquitinated peptides following digestion. **(C)** Tryptic digestion of cell lysates leaves a GlyGly remnant at sites of ubiquitination. The diGly antibody recognizes this K-ε-GG epitope and enriches lysine-linked ubiquitinated peptides. **(D)** The UbiSite antibody binds the longer ubiquitin remnant following digestion with the protease Lys-C, enriching substrates regardless of the residue ubiquitin is conjugated to. Purified N-terminal-, cysteine-, serine-, threonine-, and lysine-linked ubiquitinated peptides are processed by trypsin to generate peptides of suitable length for mass spectrometry analysis. **(E)** The antibody-free approach for ubiquitination profiling (AFUP) blocks free ε-amine groups of lysine residues with formaldehyde, followed by cleavage of ubiquitin by deubiquitinating enzymes. The newly exposed ε-amine groups at lysine ubiquitin sites are labelled by NHS-SS-biotin and enriched with streptavidin beads.

The conventional method for studying the global ubiquitinome is mass spectrometry-based proteomics, which has proven a valuable tool in mapping the canonical ubiquitin landscape (Li et al., 2021; Steger et al., 2022). Briefly, protein extracts from cells or tissues are digested using proteases to produce peptides. Due to the low abundance of ubiquitin modified proteins in the total protein pool and the transient nature of ubiquitination, most methods enrich for modified substrates, either on a protein- or peptide-based level (done prior to or post-digestion respectively). Following this, high-throughput liquid chromatography coupled to tandem MS (LC-MS/MS) is used for the identification of substrates and ubiquitin sites (Figure 2A).

### 3.1 Enrichment strategies for canonical ubiquitination

#### 3.1.1 Purifying ubiquitin substrates at a protein-level

Enrichment methods for ubiquitin substrates prior to digestion into peptides can involve purification of both exogenously expressed or endogenous ubiquitin.

##### 3.1.1.1 Exogenous

A common strategy for exogenous expression of ubiquitin is fusion of a 6/8/10xHis-tag, Strep-tag, or other epitope-tag to the N-terminus of ubiquitin. Substrates modified by tagged ubiquitin can subsequently be enriched from cell lysates using Ni-NTA, Streptavidin or anti-epitope antibody conjugated beads. These experiments can be relatively straightforward and do usually not require costly resources. A critical issue, however, is inactivation of DUBs via the use of inhibitors or denaturing lysis buffers to prevent deconjugation. Further, it can be difficult to retain similar expression levels of exogenous affinity-tagged ubiquitin compared to endogenous ubiquitin, which may alter the cellular dynamics.

##### 3.1.1.2 Endogenous

Endogenously tagged ubiquitin systems such as the Strep-tag II-ubiquitin or the StUbEx system have been developed to overcome expression challenges of exogenous approaches (Akimov et al., 2014; Kliza et al., 2017; Akimov et al., 2018a). However, tags may affect ubiquitin conjugation and deconjugation rates, thereby introducing artefacts. This is addressed by methods that utilize anti-ubiquitin antibodies or ubiquitin-binding entities. There are several anti-ubiquitin-antibodies commercially available that bind ubiquitin or specific ubiquitin-linkages, enabling purification of endogenous ubiquitinated substrates (Figure 2B) (Newton et al., 2008). A disadvantage of this approach is that using large amounts of antibody might become costly. Lastly, ubiquitin-binding domains bind ubiquitin independent of the linkage origin, which is exploited by several techniques to enrich ubiquitinated proteins from cell extracts (Figure 2B). The use of multiple ubiquitin binding domains, such as tandem-repeated ubiquitin-binding entities (TUBEs), has greatly improved the affinity, however, these domains often display a bias towards poly-ubiquitinated substrates over mono-ubiquitinated substrates (Hjerpe et al., 2009; Akimov et al., 2011; Yoshida et al., 2015; Gao et al., 2016; Scott et al., 2016; Mattern et al., 2019). Upon digestion of the enriched proteins using any of these approaches, the majority of the peptides will likely be unmodified. The presence of a large number of non-ubiquitinated peptides might hinder identification of less abundant modified peptides by mass spectrometry analysis. Additionally, most of these approaches provide limited information on the actual ubiquitin site.

#### 3.1.2 Purifying ubiquitin sites at a peptide-level

Following digestion of the proteome or a subset of enriched proteins, ubiquitinated peptides can be directly purified from the digested peptide pool. Approaches can be classified in antibody-based or antibody-free methods.

##### 3.1.2.1 Antibody-based methods

**FIGURE 3:**
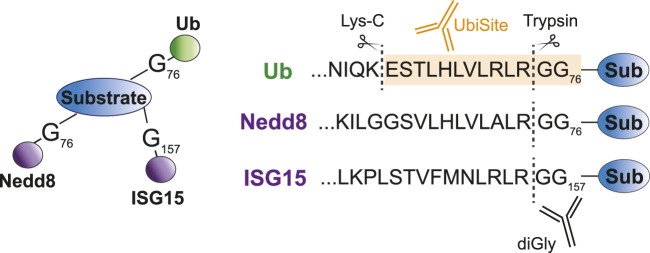
Specific recognition of ubiquitin substrates by UbiSite antibody. Ubiquitin, Nedd8 and ISG15 are conjugated to substrates through their C-terminal glycine residue. DiGly antibodies bind to the GlyGly remnant left at the site of modification following trypsin digestion and thus recognize both ubiquitin as Nedd8 and ISG15 modified peptides. The UbiSite antibody recognizes the larger remnant left by Lys-C digestion, which is specific for ubiquitin.

Antibody-based methods involve the enrichment of specific remnants following digestion. Tryptic digestion of ubiquitinated proteins, for example, leaves a typical diGly remnant at the site of ubiquitination (Figure 3). Specific diGly-antibodies bind this remnant and can thus purify ubiquitinated peptides from a trypsin digested peptide pool (Figure 2C) (Xu et al., 2010). However, diGly antibodies seem to suffer from a bias towards particular amino acids surrounding the modified lysine (Wagner et al., 2012). Secondly, the ubiquitin-like modifications (UbL) NEDD8 and ISG15, leave identical diGly remnants at their site of modification following trypsin digestion. Akimov et al. addressed this drawback with the design of the monoclonal antibody UbiSite, which recognizes the specific Lys-C induced peptide fragment of ubiquitin-modified sites, ensuring purification of ubiquitinated peptides only (Figure 3) (Akimov et al., 2018b). Following UbiSite enrichment, the Lys-C digested peptides are further processed by trypsin to generate shorter fragments, which allows for mass spectrometry analysis and mapping of ubiquitin sites (Figure 2D).

Box 1 **Phosphoribosyl-linked serine ubiquitination by *Legionella pneumophila.***

Pathogens have developed sophisticated strategies for infiltrating host cells to ensure effective replication and transmission. One of their key characteristics involves secreting pathogenic effectors into host cells that enable pathogens to manipulate molecular pathways of the cell. A prime example of this involves hijacking the host ubiquitin system through unconventional ubiquitination by secreted effectors of *L. pneumophila*: the SidE effector proteins*.* Members of this family (SdeA, SdeB, SdeC and SidE) catalyze phosphoribosyl-linked (PR) serine ubiquitination, which has been shown to remodel the ER and Golgi, thereby promoting infectivity of *L. pneumophila* (Kotewicz et al., 2017; Liu et al., 2021). Interestingly, PR-ubiquitination is highly distinct from endogenous canonical ubiquitination as it does not rely on the typical E1, E2, E3 enzymatic cascade but is mediated by a single enzyme from the SidE family (Qiu et al., 2016). Moreover, SidE effector proteins conjugate ubiquitin not through its C-terminal Gly76 but conjugate ubiquitin’s Arg42 to substrate hydroxyl groups via a phosphoribosyl linker. SidE members harbor an ADP ribosyl transferase (ART) domain that ADP-ribosylates ubiquitin by transferring ADPR from NAD^+^. Following this, the phosphodiesterase (PDE) domain of SidE members recognize this intermediate and forms a phosphodiester bond between phospho-ribosylated (PR) ubiquitin and a substrate serine residue (Figure 1C) (Bhogaraju et al., 2016; Akturk et al., 2018; Kalayil et al., 2018).

PR-ubiquitination by SidE effectors targets a diverse array of host proteins, including Golgi-, mitochondria-, ER- and autophagy-associated proteins (Bhogaraju et al., 2016; Qiu et al., 2016; Kotewicz et al., 2017; Wan et al., 2019; Shin et al., 2020; Liu et al., 2021). Similar to regulation of canonical ubiquitination, modification of host proteins is reversible, albeit conventional DUBs cannot process PR-linked ubiquitination. Deconjugation is achieved by specific PR-ubiquitin erasers, DupA and DupB, secreted by *L. pneumophila* itself (Wan et al., 2019; Shin et al., 2020). Moreover, *L. pneumophila* also regulates the extent of PR-ubiquitination by encoding for the effector SidJ, a glutamylase that can block activity of SdeA by attacking the catalytic glutamate located in the ART domain (Bhogaraju et al., 2019; Black et al., 2019; Gan et al., 2019; Sulpizio et al., 2019). This highlights the sophisticated toolkit *L. pneumophila* has evolved to hijack host cellular processes with a chemically and mechanistically distinct form of ubiquitination.

##### 3.1.2.2 Antibody-free methods

Two methods provide an alternative to antibody-based enrichment: combined fractional diagonal chromatography (COFRADIC) and antibody-free approach for ubiquitination profiling (AFUP). These methods start with chemical blockage of free amines from unmodified lysine residues. Subsequently, ubiquitin molecules are hydrolyzed by USP2 to expose amines, which are then labelled, either by Gly-BOC tags (COFRADIC) or NHS-SS-biotin (AFUP). In the case of COFRADIC, the BOC groups are removed by trifluoracetic acid leaving ubiquitin sites marked by a Gly residue, which can be identified by mass spectrometry data analysis (Gevaert et al., 2002; Stes et al., 2014). The less time- and resource intensive AFUP method utilizes streptavidin beads to selectively enrich the ubiquitinated peptides (Figure 2E) (Sun et al., 2023). Overall, the antibody-free approaches appear less effective with fewer ubiquitin sites identified compared to antibody-based methods. Lastly, the innovative Ub-clipping method allows for the investigation of ubiquitin chain architecture in addition to identifying substrates and sites. Ub-clipping utilizes an engineered viral protease that cleaves di-ubiquitin moieties after Arg74, leaving the signature C-terminal GlyGly peptide attached to the modified residue. This preserves ubiquitin chain architecture information that is subsequently deciphered by intact mass analysis of the generated monoubiquitin species (Swatek et al., 2019).

### 3.2 Enrichment strategies for non-canonical ubiquitination

The lower stoichiometry of ubiquitinated proteins compared to their unmodified counterparts is challenging for mass spectrometry. Non-canonical ubiquitination events are expected to be even sparser in the proteome than lysine-directed ubiquitination events. Additionally, the labile nature of thioester and oxyester bonds render mapping the non-lysine ubiquitinome even more demanding. Conventional sample preparation techniques need to be restricted in the use of high temperature, high or low pH and reducing agents to avoid hydrolysis of ester bonds.

#### 3.2.1 N-terminal ubiquitination

Most enrichment methods primarily enable purification of lysine-linked ubiquitin conjugates. However, recently four monoclonal antibodies were developed that specifically target the N-terminus linked diGly remnant. These recognize the ubiquitin-α-amino peptide bonds, allowing for enrichment of N-terminally ubiquitinated proteins (Davies et al., 2021). Similarly, the UbiSite antibody is not restricted to lysine ubiquitination as the epitope recognizes a fragment solely within ubiquitin itself (Akimov et al., 2018a). The previously described StUbEx PLUS is an antibody-free approach that can also be used to purify N-terminally ubiquitinated substrates as it has no preference for either lysine or N-terminal ubiquitin (Akimov et al., 2018b). Using these approaches, the authors have detected 109 (Davies et al., 2021), 104 (Akimov et al., 2018a) and 72 (Akimov et al., 2018b) N-terminally modified proteins, respectively. Most substrates were uniquely identified in one of the three studies, with 22 proteins identified in at least two studies, and only EEF2 and TXNL1 commonly detected across all three studies (Table 2). While this naturally could be attributed to the different cell types used in these studies, it could also suggest that these approaches still have limitations in comprehensively capturing the N-terminal ubiquitinome.

**TABLE 2:** List of common N-terminally ubiquitinated proteins identified (X) in at least two out of three proteomic studies that included enrichment of N-terminal modified substrates.

Genes	Davies et al. (2021)	Akimov et al. (2018a)	Akimov et al. (2018b)
ALDOA		X	X
ATIC	X	X	
CCT3	X		X
DUT		X	X
EEF2	X	X	X
EIF3G		X	X
FAM96B	X		X
FKBP1A		X	X
GADD45A		X	X
KRIT1		X	X
B4GALNT2	X		X
PAPOLA		X	X
RPS7	X	X	
SERBP1		X	X
SLX1A	X		X
STAM		X	X
STAM2		X	X
SURF4		X	X
TMEM259	X		X
TRAPPC1	X		X
TXNL1	X	X	X
UCHL5	X	X	

#### 3.2.2 Non-lysine ubiquitination

To our knowledge, purification of non-lysine linkages for LC-MS/MS analysis has thus far only been described using the UbiSite antibody. The UbiSite antibody recognizes an internal C-terminal sequence of ubiquitin, expanding its applicability beyond canonical ubiquitination (Akimov et al., 2018a). Following UbiSite peptide purification of a targeted sample, MS/MS spectra of the fragmented ions evidently pinpointed five serine and threonine ubiquitination sites on three HOIL-1 substrates and additionally identified serine and threonine-linked ubiquitin chains (McCrory et al., 2022).

### 3.3 High throughput protein identification using LC-MS/MS

Once the sample of interest is digested into a complex peptide mixture, peptides can be separated by liquid chromatography followed by analysis using mass spectrometry. LC-MS/MS systems use two primary modes of data acquisition: data dependent acquisition (DDA) or data independent acquisition (DIA) (Venable et al., 2004; Bilbao et al., 2015; Hu et al., 2016; Zhang et al., 2020). DDA, which is most commonly employed, selects the most abundant peptides from the first round of MS and sends these precursor ions for fragmentation and a second round of MS. Protein identification is deduced from the MS/MS spectra using search algorithms that often rely on spectral comparison. DDA is thus biased towards abundant peptides and may miss low abundant peptides. In the DIA mode, all ions within a selected m/z range are fragmented and analyzed in a second round of MS. Analyzing DIA acquired spectra is more complicated due to the highly complex nature of fragmented ion spectra. Protein identification of these spectra often relies on database-based search engines using a pre-existing library of MS1 spectra. Existing DIA libraries do not yet include ubiquitinated peptides, but a library can be built beforehand with deep DDA acquisition. Of note, software for analysis of DIA data, like Spectronaut and DiaNN, has developed tremendously over the last few years allowing similar high-quality analyses using library-based or library-free approaches (Demichev et al., 2020; Trulsson et al., 2022).

Various software tools are available which have integrated database-searching engines that subsequently allow for identification of peptides from DDA MS data, such as Mascot (Perkins et al., 1999), MaxQuant (Cox and Mann, 2008), PEAKS studio (Zhang et al., 2012) and SEQUEST (Eng et al., 1994). These search engines use algorithms to calculate predicted spectra for all peptide sequences and compare them to the given spectra of a peptide and its fragments. Targeted instructions allow search engines to identify peptides with specified modifications such as ubiquitin. The diGly remnant left at the site of ubiquitination following tryptic digest, introduces a mass shift. GlyGly modified peptides will have a mass difference of 114.0429 Da compared to the unmodified peptide, which can be added to the search parameters and allows for determination of the site of ubiquitination.

Substantial development in purification strategies, mass spectrometry sensitivity and data acquisition methods have led to major progress in mapping the canonical ubiquitinome with site-specific resolution. Currently, studies have been able to identify approximately 42,000 (Akimov et al., 2018a), 63,000 (Akimov et al., 2018a; Trulsson et al., 2022) and 90,000 (Hansen et al., 2021) sites using the StUbEX PLUS, UbiSite and diGly approaches, respectively. Methodologies for identification of non-canonical ubiquitination are not yet as advanced as those used for canonical ubiquitination. Approaches utilizing UbiSite purification have shown promise with the identification of endogenous non-lysine ubiquitinated substrates in a target sample. However, to perform a global and comprehensive analysis of the non-canonical ubiquitinome, there is a need for further enhancement of mass spectrometry methods specifically tailored to detect these low-abundant, labile, ester-linked ubiquitination events.

## 4 Non-canonical ubiquitination: future considerations

Since its discovery, the number of identified substrates and components of the ubiquitin machinery involved in the generation and breakdown of uncommon linkage types has steadily increased. Regardless, the non-lysine ubiquitination research still is in its infancy and countless sites and modulators have presumably remained undetected owing to the lack of suitable analytical methods and chemical tools. The appearance of thio- and oxyester-linked ubiquitination as a viable addition to the lysine ubiquitin code has furthermore raised questions concerning interplay with canonical ubiquitination, PTM competition and potential crosstalk (e.g., with phosphorylation and ADPribosylation). (Poly)-ubiquitin chains linked via serine or threonine are especially intriguing and have the potential to drastically expand the ubiquitin signaling landscape. They might present novel avenues for regulating essential cellular pathways, although exact functions and mechanisms of action remain largely speculative to date. Oxyester-linked ubiquitination, for instance, could function as a stop signal to restrict the size of isopeptide-anchored ubiquitin or even terminate chain extension in a negative feedback loop (Kelsall et al., 2019; Petrova et al., 2021). Past investigations have indicated that the same ubiquitination machinery is capable of modifying both non-lysine and lysine residues (Wang and Subramani, 2017), which might suggest that, in some cases, the position of the amino acid rather than its nature is potentially dictating its ubiquitination fate. This contrasts other reports, which showcase zero-tolerance in deviating from lysine as acceptor in ubiquitin-chain formation (Liwocha et al., 2021). It will therefore be crucial to assess the competition of oxyester-linked ubiquitination and canonical lysine modification in the context of versatility and promiscuity of processing enzymes. This could be particularly relevant for threonine- and serine-linked ubiquitin chains, as the participating residues Thr12, Thr14, Ser20, and Thr22 are in close proximity to the conventional lysine residues (McCrory et al., 2022). Oxyester-linked ubiquitination of these amino acids could also be involved in a direct competition with phosphorylation, as almost all hydroxyl containing side chains of ubiquitin were shown to be phosphorylated (Herhaus and Dikic, 2015; Swatek and Komander, 2016; Hepowit et al., 2022) and it cannot be excluded that they might also be susceptible to oxyester-linked ubiquitin chain formation. As a result, phosphorylation of these residues could regulate ubiquitin chain formation by preventing the attachment of another ubiquitin unit or *vice versa*. Finally, a growing body of reports shows that non-proteinaceous ubiquitination substrates such as carbohydrates, lipo-poly-saccharides, ADP-ribose and phospholipids (reviewed in (Squair and Virdee, 2022; Dikic and Schulman, 2023; Sakamaki and Mizushima, 2023) also rely on oxyester linkages being forged between ubiquitin and substrates.

### 4.1 Towards a comprehensive understanding of non-canonical ubiquitination: emerging questions

The current state of research leaves no doubt that the non-canonical ubiquitinome is far from being unraveled. While existing high-throughput technologies such as the UbiSite approach (Akimov et al., 2018a) might be suitable to detect and assign non-canonical ubiquitination sites, other methods will have to be developed or adapted to address the inherent challenges that accompany labile PTMs. Mass spectrometry is undoubtedly the gold standard for identifying protein ubiquitination sites (Steger et al., 2022), however, standard MS workflows might be detrimental for these labile modifications that are typically very sensitive to heat and pH value. Moreover, evidence of non-lysine ubiquitination might be lost if not specifically looked for by software packages. Future MS-based ubiquitin studies should thus take these considerations into account, since non-canonical ubiquitination could be a modification more abundant than previously considered.

The tightly regulated interplay between E1, E2, E3 and DUB enzymes is a fundamental characteristic of the complexity of the ubiquitin code. It is thus essential to disentangle the molecular determinants that steer esterification and esterase activity and disclose the key players involved in these processes. As for E2s, UBE2W and UBE2J2 are the only enzymes known to display non-lysine activity, where UBE2J2 even prefers to ubiquitinate hydroxyl containing side chains in the presence of lysine residues (Wang et al., 2009; De Cesare et al., 2021). Intriguingly, both lack the canonical HPN motif involved in catalysis and a conserved downstream aspartic acid/serine residue, which is supposed to lower the pKa of the substrate lysine during iso-peptide bond formation (Yunus and Lima, 2006; Plechanovová et al., 2012; Valimberti et al., 2015). Hence, this structural feature could be indicative of non-lysine activity and would also include UBE2Q1 and UBE2Q2, two poorly characterized E2s that also lack the abovementioned structural features. MYCBP2 constitutes the first example of an RCR (RING-Cys-Relay) E3 ligase and, strikingly, prefers ubiquitination of threonine residues, while being virtually inactive against lysine substrates (Pao et al., 2018; Mabbitt et al., 2020). The crystal structure reveals that this selectivity might be due to a hydrophobic pocket in the esterification site that optimally accommodates the β-methyl group of threonine. More structural data of E3 ligases will help to explain whether this unique RCR mechanism is a hallmark of esterification activity. Interestingly, a considerable number of DUBs is able to cleave both isopeptide- and oxyester-linked ubiquitin from substrates, while the hydrophobic active site of MJD family DUBs appears to confer a selective esterase activity (Sun et al., 2018; De Cesare et al., 2021; Szczesna et al., 2023). Although the analyzed substrates were non-physiological, the presence of multiple DUBs that preferentially deubiquitinate oxyester-linked substrates points towards non-lysine ubiquitination being a relevant cellular modification rather than a biological oddity. Furthermore, the fate of ubiquitinated substrates is dependent on “readers”: proteins that are recruited to ubiquitinated substrates and enable the execution of distinct cellular responses. So far, no ubiquitin binding domain that specifically recognizes non-lysine ubiquitin linkages has been discovered. However, the idea of ubiquitin readers capable of discriminating lysine and non-lysine modifications is captivating.

To fully understand its role in a cellular and biological context, it will be indispensable to identify interactors of thioester- or oxyester-linked ubiquitin and potentially disclose ester-specific ubiquitin binding domains. The (bio)chemical means to (semi)synthesize full-length ubiquitin and modified analogues has tremendously advanced the field and helped to unravel some of the molecular mechanisms of ubiquitination (El Oualid et al., 2010; Kumar et al., 2010; Mulder et al., 2016; Huppelschoten and van der Heden van Noort, 2022; Hehl et al., 2023). Consequently, many different probes have been employed to examine the ubiquitinome and broaden the scope of known interactors (Zhang et al., 2017; Zhao et al., 2017). To circumvent any stability issues and prevent premature DUB cleavage, the linkage site is typically modified to harbor a non-hydrolyzable chemical entity. Readers of non-lysine ubiquitination could be identified analogously, utilizing non-hydrolyzable serine-, threonine-, or cysteine-linked probes and chemical proteomics. These compounds could also be used to raise linkage-specific antibodies in the long run, which would be an invaluable addition to the toolbox. While non-hydrolyzable ubiquitin baits target interacting proteins through their binding affinity, DUBs can also be profiled by their catalytic activity using activity-based probes (ABPs) (Hewings et al., 2017; Conole et al., 2019; Huppelschoten and van der Heden van Noort, 2022). Over the last decade, many ubiquitin-derived tools were generated to identify, characterize, and annotate specific DUB activity (Iphöfer et al., 2012; Ekkebus et al., 2013; McGouran et al., 2013; Li et al., 2014; Mulder et al., 2014; Flierman et al., 2016; Geurink et al., 2019; Szczesna et al., 2023) opening the way to detect and validate non-canonical linkage-specific DUBs.

Non-canonical ubiquitination has the potential to evolve into a substantial component of the ubiquitin code and might play a vital role in cellular signaling and homeostasis. It contributes to the ever-growing complexity of the ubiquitin machinery and is likely involved in crosstalk with conventional isopeptide-linked ubiquitination and other PTMs. The established analytical, chemical and biological methods and procedures to study lysine-directed ubiquitination provide an excellent springboard for investigating non-lysine ubiquitination, if modified appropriately. The combination of specialized MS-protocols and various (bio)chemical tools will be pivotal both for identifying unknown substrates of non-lysine ubiquitination and assigning responsible conjugating, ligating and deubiquitinating enzymes to further expand our knowledge on the already dauntingly complex ubiquitin code.
